# The rough-and-tumble play of rats as a natural behavior suitable for studying the social brain

**DOI:** 10.3389/fnbeh.2022.1033999

**Published:** 2022-10-18

**Authors:** Sergio M. Pellis, Vivien C. Pellis, Jackson R. Ham, E. J. M. Achterberg

**Affiliations:** ^1^Department of Neuroscience, University of Lethbridge, Lethbridge, AB, Canada; ^2^Division of Behavioural Neuroscience, Unit Animals in Science and Society, Department of Population Health Sciences, Faculty of Veterinary Medicine, Utrecht University, Utrecht, Netherlands

**Keywords:** social play, rats, social dynamics, social skills, individual differences

Play fighting, the most commonly reported form of social play, involves competition to gain an advantage (Aldis, 1975), but there are two features that make it different from serious fighting. First, it is highly pleasurable and associated with a positive affective state (Vanderschuren et al., 2016). Second, the competition is moderated by cooperation, ensuring that the interactions have a degree of reciprocity or turn taking between partners (Palagi et al., 2016a). That is, the player with the advantage may voluntarily relinquish it, thus allowing a role reversal to occur, with the original defender gaining the opportunity to become the attacker (Pellis and Pellis, 2017). Thus, although actions performed during play fighting can be accurately described as involving attack, defense, and counterattack, the context of their use should not be confused with that of aggression. For this reason, we will refer to this kind of play as rough-and-tumble play (RTP), to highlight the cooperative aspect of these interactions. Nonetheless, competition during RTP can create ambiguity as to whether a partner may be taking unfair advantage of the situation, with effective communication being important to avoid the risk of escalating to aggression or to partners being ostracized if they play too roughly (Palagi et al., 2016b). Creating and resolving ambiguity, which requires balancing competition and cooperation, also provides a vehicle by which juveniles and adolescents can train socio-cognitive skills (Pellis and Pellis, 2009, 2017).

Laboratory rats have been an important model species with which to study the neurobiology of RTP (Siviy and Panksepp, 2011; Siviy, 2016; Vanderschuren et al., 2016). As shown in Figure 1, RTP in rats involves competition to gain access to the partner's nape of the neck, which is nuzzled with the snout if contacted (Pellis and Pellis, 1987; Siviy and Panksepp, 2011). A variety of tactics are used to attack and defend the nape, including launching counterattacks following a successful defense (Himmler et al., 2013; Pellis et al., 2022). Rats are a particularly good model species for studying RTP, as this behavior not only differs from serious fighting in the ways described above, but also because serious fighting involves attacking other body targets, namely the flanks and rump, which are bitten if contacted (Blanchard et al., 1977; Pellis and Pellis, 1987). Consequently, it can be readily discerned when a playful encounter escalates to serious fighting, as the aggressor switches from attacking the nape to biting the partner's posterior (Stark and Pellis, 2020).

**Figure 1:**
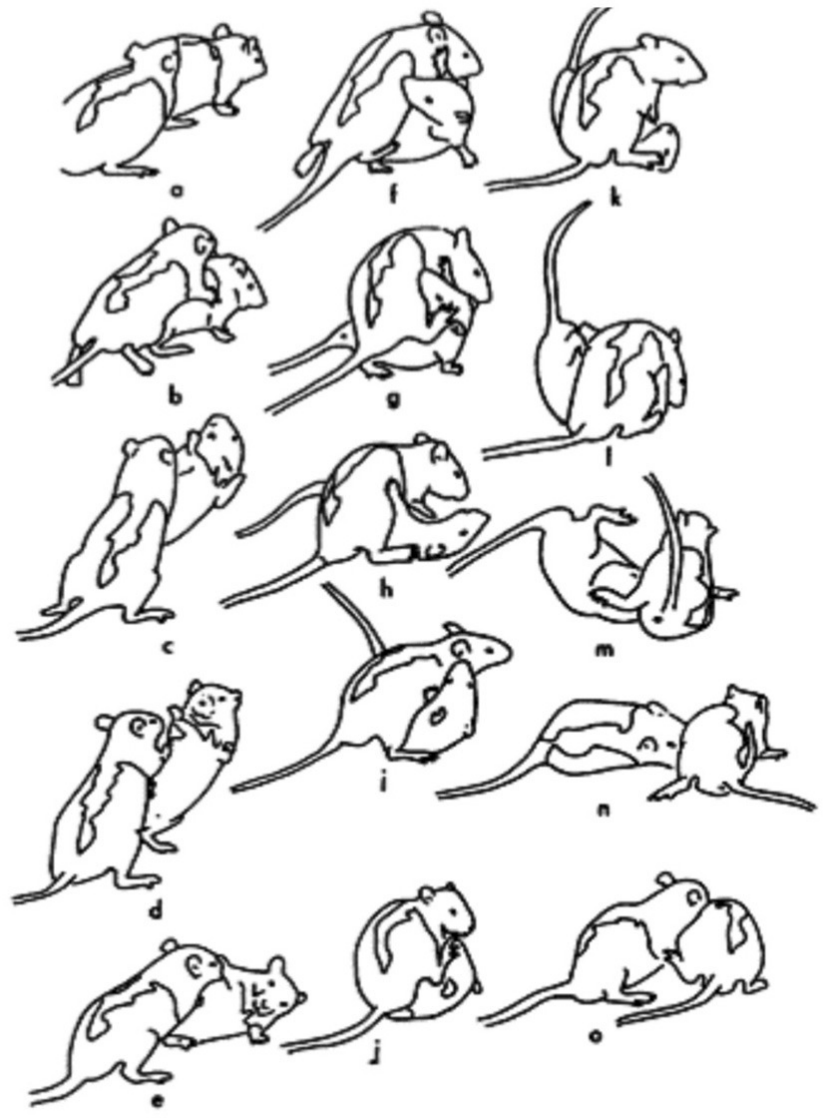
A sequence of play fighting is shown for a pair of juvenile rats. The rat on the left approaches the partner **(a)**, and reaches toward its nape from the rear **(b)**, but before contact can be made, the partner rotates around its longitudinal axis **(c)** to face its attacker **(d)**. By moving forward, the attacker pushes the defender onto its side **(e)**. The defender then rolls over onto its back as the attacker continues to reach for its nape **(f–h)**. Once in the supine position, the defender launches an attack on its partner's nape **(i)**, but fails due to its partner's use of its hind foot **(j,k)**. Eventually, the rat on top **(l)** is pushed off by the supine animal **(m)**, which then regains its footing **(n)**. The original defender then lunges toward its partner's nape **(o)**. (Reprinted from Pellis and Pellis, 1987, with permission).

In laboratory rats, RTP begins to emerge in the third week after birth, peaks in occurrence between the fourth and fifth week and then declines with the onset of puberty, but continues into adulthood, albeit at a lower level (Thor and Holloway, 1984; Pellis and Pellis, 1990, 1997). While the motivation to play can be modified by early experiences mostly derived from the mother (Parent and Meaney, 2008; Van Hasselt et al., 2012), once play begins at around 17 days of age, it takes about 10 days for the repertoire of tactics used during play to mature fully and this maturation appears to be little influenced by rearing experiences (Himmler et al., 2015).

Neither the age-typical changes in the frequency of RTP nor the availability of the full behavioral repertoire used during RTP depend on the cortex, but cortical systems, especially those of the prefrontal cortex, are critical to allow rats to modulate aspects of their playful responses, depending on their partner's actions and their identity (Pellis and Pellis, 2016). Even though both sexes and all strains of rats thus far studied share the same basic play repertoire, there are subtle differences that can be informative. For example, under some rearing and testing conditions, males initiate more nape attacks (Thor and Holloway, 1984), and in early adulthood are more likely to use defensive tactics that makes their RTP seem rougher (Pellis, 2002). Similarly, there are differences across strains in the relative frequency of nape attacks and in the use of defensive tactics that lead to evading contact or promoting close-quarter wrestling (Himmler et al., 2016). These differences have provided valuable tools for using sex and strain differences to identify the roles of specific neural systems and neural networks in regulating particular aspects of play (Siviy, 2020; VanRyzin et al., 2020).

Given that RTP is naturally occurring, no experimental training is needed to teach the animals to play. Moreover, as rats have a degree of sophistication in their RTP that is comparable to that of many primates and other social mammals (Pellis and Pellis, 2017), they are an ideal laboratory species to study not only play, but also, by extension, aspects of the social brain that make play possible. For example, the development of the medial prefrontal cortex (mPFC) and associated socio-cognitive skills is influenced by the experience of RTP with peers in the juvenile period (about 28–40 days post birth; Bell et al., 2010; Baarendse et al., 2013; Schneider et al., 2016), and the mPFC has a crucial role in coordinating actions with partners as juveniles and as adults in both playful and non-playful social interactions (Bell et al., 2009; Van Kerkhof et al., 2013; Himmler et al., 2014; Stark and Pellis, 2020). That is, social play is a valuable window into the social brain. Two recent developments illustrate the opportunities provided by the study of play in rats.

## Individual differences and the drivers of play

Even within members of the same sex and same strain, not all individuals play to the same degree—some rats consistently play more than others (Lampe et al., 2017; Lesscher et al., 2021). Such individual differences provide an opportunity to refine the search for the neural mechanisms that regulate play. For instance, attack and defense during RTP tend to involve independent mechanisms (Himmler et al., 2016). Within a strain, high players not only initiate more nape attacks, but also preferentially use a different suite of the rat-typical defensive tactics than do low players (Pellis et al., 2022). As the use of ultrasonic vocalizations (USV) can be critical for communication during play, facilitating role reversals and avoiding escalation to aggression (Kisko et al., 2017; Burke et al., 2020), rats with different styles of RTP may need to modify their use of such calls (Pellis et al., 2022) to play together effectively, making RTP in rats a useful window into subtle social communication processes.

There is strong evidence that the difference in launching nape attacks is linked to differences in mesolimbic dopamine activity (Vanderschuren et al., 2016; Siviy, 2020). That mesolimbic dopamine activity does not just affect the launching of nape attacks, but also the motivation to engage in play, has been shown by operant conditioning methods, in which dopamine manipulations affect how hard rats will work for the reward of access to a playmate (Achterberg et al., 2016). While opioid systems have been implicated in the rewards derived from engaging in play (Vanderschuren et al., 2016; Achterberg et al., 2019), exactly which neural circuits are associated with what types of playful actions and styles of play remains to be investigated. In addition, how cortical and subcortical neural circuits that are known to be involved in regulating affective and aversive USV (Brudzynski, 2013) are modulated to accommodate different styles of play remains to be determined. However, attempts to explain the neural mechanisms associated with individual differences in RTP have started to emerge in the literature (e.g., Reppucci et al., 2020).

## Group dynamics and partner choice

Initial studies of play in rats involved observing them in their home enclosures with the whole litter present to assess the occurrence of RTP (Baeninger, 1967; Meaney and Stewart, 1981; Pellis and Pellis, 1983). Such a collective paradigm, although naturalistic, makes testing the effects of specific treatments on play nearly impossible. Consequently, a dyadic paradigm was developed—rats are tested in pairs in an enclosure to which they have been habituated following a period of social isolation to increase their motivation to engage in play (Panksepp and Beatty, 1980; Panksepp, 1981). This dyadic paradigm has now become the most widely used experimental paradigm for testing RTP in rats (Pellis et al., 2022). Variations on the theme include the length of pre-test social isolation and whether experimental rats are tested with a same condition partner, an untreated partner, or both. In addition, rats can be partnered either with a familiar rat or a stranger. As the rats in the dyadic paradigm are tested for a fixed duration (5–20 min being most common), the effects on both the overall amount of play, as measured by the number of nape attacks launched, and the style of play, as measured by the frequency of use of the different defensive tactics, can be compared between experimental and control pairs. This level of control is especially important for pharmacological manipulations, as the animals need to be tested when the drug reaches its peak effects on the brain (e.g., Field and Pellis, 1994; Achterberg and Vanderschuren, 2020). However, the downside to the level of control achieved by the dyadic paradigm is that the rats lose the ability to choose their play partner, as it is the experimenter who selects the partner.

Not all partners are equally attractive as play mates (Holloway and Suter, 2004; Pellis et al., 2006). When rats are tested in groups, in which multiple partners are available, play is not distributed evenly. Rather, some partners are favored over others, and this is not simply a by-product of which of the animals are in closest proximity—rats will leave the company of animals in one part of the enclosure and travel to the other side to initiate play with a particular animal (Pellis et al., 2022). That is, some potential play partners are preferred over others. Experimental manipulations may alter the ability of rats to discriminate between partners (Pellis et al., 2006), and this may go undetected in the standard, dyadic play paradigm, in which interaction with only one partner is possible, potentially leading to the false conclusion that the experimental manipulation has no effect as the amount of play is the same as that of the control. Thus, multi-animal paradigms are needed to offset the disadvantages that have come with the advantages gained from the dyadic paradigm. Indeed, more sophisticated methods are becoming available for continuous recording and scoring of behavior in a home cage (Greico et al., 2021). A combination of dyadic testing, enabling more control in how a particular rat deals with a particular partner, and home cage, group testing, allowing the animal to exercise control over with whom to play and where, will capture the advantages of both approaches, and so assess a deeper analysis of the effects of treatments on social behavior.

## Combining naturalistic observations with experimental manipulations

As indicated by the two examples above, so-called outliers in naturally occurring behavior like RTP can be highly informative about underlying biological processes, rather than, as is typically the case in many contrived experiments, being viewed as noise that interferes with the interpretation of results and future replication. However, while outliers can focus our attention on phenomena that may be missed when comparing group means, some degree of experimental control over the behavior may be needed to identify what is most relevant to the animals. That is, naturalistic observations and experimental manipulations can be used together in an iterative manner. An example will illustrate this synergy.

In a group setting, rats preferentially play with particular partners (Pellis et al., 2022), but what makes one partner more attractive than another? As indicated above, some rats are more playful than others and also have differing styles of play. Therefore, one possibility is that rats seek out partners with congruent styles of play. If this is so, this could be tested in a dyadic setting by matching congruent and incongruent pairs. However, this is still contaminated by the fact that the rat has no choice but to play with the partner available—and in such a context, both partners may need to make compromises in how they play. Another approach is to allow the subject to play with rats having different play styles, and then give the subject a choice in an operant conditioning paradigm. If play style is important, then the rat should be willing to work harder to access the partner with their preferred style. Similarly, other features of potential partners could be tested.

Studying RTP can be a valuable tool both for basic research on communication and other processes that are involved in regulating social behavior (Palagi et al., 2016b), and for translational research on neurodevelopmental disorders (Burke et al., 2017). Indeed, identifying that turn taking, as shown by the occurrence of role reversals, is a key feature of RTP that promotes the development of socio-cognitive skills (Pellis et al., 2019; Stark et al., 2021), has been important for engineering therapeutic play contexts that similarly promote the development of those skills in children (e.g., Diamond et al., 2007; Nijhoff et al., 2018).

## Author contributions

SP, VP, JH, and EA: conceptualization and writing. All authors contributed to the article and approved the submitted version.

## Funding

Many of the ideas expressed arose from research supported by the following two grants: Natural Science and Engineering Council of Canada (NSERC), Grant Code: 2018-03706; Dutch Research Council Domain Science (Nederlandse Organisatie voor Wetenschappelijk OnderzoekŮdomein Exacte en Natuurwetenschappen, NOW-ENW), Grant Code: 016.Veni.181.039.

## Conflict of interest

The authors declare that the research was conducted in the absence of any commercial or financial relationships that could be construed as a potential conflict of interest.

## Publisher's note

All claims expressed in this article are solely those of the authors and do not necessarily represent those of their affiliated organizations, or those of the publisher, the editors and the reviewers. Any product that may be evaluated in this article, or claim that may be made by its manufacturer, is not guaranteed or endorsed by the publisher.
